# Drag Reduction Mechanism of Bionic Riblets and Their Application on Centrifugal Fan Blades

**DOI:** 10.3390/biomimetics11100694

**Published:** 2026-09-29

**Authors:** Xiaopei Yang, Bin Zuo, Qianhao Xiao

**Affiliations:** 1 School of Mechanical and Electrical Engineering, Suzhou Polytechnic University, Suzhou 215104, China; 2 Jiangsu Province Robot and Intelligent Equipment Engineering Technology Research and Development Center, Suzhou 215104, China; 3 School of Energy and Power Engineering, Changsha University of Science and Technology, Changsha 410114, China

**Keywords:** bionic, riblets, large eddy simulation, flow separation, drag reduction, backward centrifugal fan, efficiency improvement

## Abstract

Bio-inspired riblet technology has progressed in recent years in suppressing near-wall flow separation and reducing drag. However, significant benefits are obtained only when the riblet geometry matches the local flow conditions. Further study is still required before riblets can be reliably applied to low-speed fan blades, whose flow fields are inherently complex. In the present work, large eddy simulation (LES) is used to examine how riblets oriented perpendicular to the flow direction influence the aerodynamic performance and flow characteristics of an airfoil typical of low-speed turbomachinery. Riblets with various groove widths and heights are designed, and the effect of their placement along the airfoil suction surface (SS) is also investigated. The friction coefficient, pressure coefficient, and near-wall velocity distributions of the different configurations are compared. Based on the friction coefficient, the airfoil SS can be divided into three regions: a rapidly decreasing region (RDR) near the leading edge, a stable region (SR) in the middle, and a fluctuating region (FR) at the trailing edge. It is found that riblets of different sizes placed within the RDR can suppress vortex development and improve the near-wall flow. As a result, the RDR exhibits better adaptability to riblet parameters than the SR and FR. Motivated by these findings, riblets were applied to the blades of a backward centrifugal fan, specifically within the blade RDR, with a uniform width and height of 0.5 mm. Fan test results demonstrate that riblets covering 10–20% of the blade camber line increase the fan efficiency by 4.5% and the total pressure by 4.2%, respectively. The aerodynamic performance of the fan under low-flow conditions is markedly improved.

## 1. Introduction

Over billions of years of natural evolution, living organisms have developed remarkable survivability and environmental adaptability [1,2,3]. Researchers have identified anatomical features in numerous species that enhance lift and reduce motion resistance [4,5], offering valuable design references for decreasing the resistance of fluid equipment and improving its operational efficiency [6]. Inspired by the rough skin of sharks, it has been hypothesized that a non-smooth surface can reduce wall friction, which led to the concept of riblets, a surface textured with microscopic grooves. In engineering applications, the complex three-dimensional morphology of shark skin is often simplified into regular riblet profiles, such as triangular or V-shaped grooves, to retain the main ridge-groove characteristics while facilitating geometric control and fabrication [7]. The riblets was first examined on a flat plate, confirming a measurable drag reduction effect [8].

Many studies have since explored the underlying drag reduction mechanisms. It was initially proposed that riblets reduce drag by weakening momentum exchange induced by near-wall vortices in the turbulent boundary layer [9,10]. On one hand, counter-rotating vortex pairs have been observed on the grooves, which attenuate the longitudinal vortices and suppress the formation of spanwise low-velocity streaks [11]; these findings were subsequently reinforced by further investigations [12,13]. On the other hand, the ‘protrusion height’ [14] concept based on viscosity theory provides another prominent explanation: because the velocity gradient at the groove bottom is nearly zero, the riblets create an equivalent smooth wall, and the velocity gradient at this equivalent wall is smaller than at the original surface, thereby reducing friction drag [15].

In addition to the underlying flow mechanism, riblet performance is strongly affected by its geometry and the local flow conditions. Previous studies have shown that the drag reduction effect depends on the riblet cross-sectional shape [16,17], the geometric dimensions, and the corresponding flow conditions [18]. Table 1 summarizes reported riblet performances under various flow conditions, showing that the level of drag reduction varies considerably with flow velocity and the shape of the wall surface on which the riblets are placed. For instance, sawtooth riblets of 0.1 mm applied to a wind turbine airfoil [19] and to an NACA4412 airfoil [20] at similar Reynolds numbers exhibit markedly different effectiveness. Nevertheless, when the riblet geometry is highly compatible with the flow conditions, the airfoil drag can be reduced and the lift coefficient increased; the lift-to-drag ratio has been reported to rise by up to 323% [21].

To better characterize the drag reduction effect of riblets in different flow conditions, researchers have introduced dimensionless geometric parameters: the dimensionless height *h*^+^ and dimensionless spacing *s*^+^. Walsh [28] found that riblets with *h*^+^ < 25 and *s*^+^ < 30 can effectively reduce the friction drag of a flat plate. However, the optimal riblet dimensions for achieving the maximum drag reduction remain a subject of debate [29]. Mawignon et al. [30] suggested that a greater drag reduction is obtained when the *s*^+^ is between 8.5 and 25, with a height *h*^+^ of 5.4.

Precision surface modifications on turbomachinery blades, such as the addition of small local surface structures, can effectively suppress flow separation [31,32,33]. Encouraged by the well-established drag reduction effect of riblets on flat plates and airfoils [34], researchers have attempted to apply them to turbomachinery blade surfaces in order to improve aerodynamic performance [35,36]. Abbasi et al. [37] applied riblets to the blades of a Wells turbine and observed a 2.3% increase in efficiency. Li et al. [38] achieved a 20.5% reduction in total pressure loss in a compressor cascade. These studies demonstrate the potential of riblets for turbomachinery applications. However, their performance remains highly sensitive to geometric parameters, surface location, and local flow conditions.

Previous studies on transverse riblets have mainly focused on specific geometries or individual surface locations, while the combined effects of chordwise position and riblet size on a transitional airfoil have received less attention. In addition, the applicability of riblet configurations identified from fixed-airfoil studies to rotating turbomachinery has not been sufficiently examined. These limitations make it difficult to directly determine suitable riblet parameters for low-speed fan applications. Consequently, further investigation into the drag reduction mechanisms and practical deployment of riblets is still needed.

This study focuses on the drag reduction mechanism of riblets oriented perpendicular to the flow direction. Large eddy simulation (LES) is first used to evaluate the combined effects of riblet position and size on aerodynamic performance of a quasi-three-dimensional airfoil and to analyze the associated changes in vortical structures. The sensitivity of the airfoil suction surface to riblet parameters and the suitable riblet locations and geometric ranges are identified. The selected configuration is then applied to the blades of a backward centrifugal fan and evaluated experimentally, thereby extending the fixed-airfoil analysis to rotating fan conditions.

## 2. Calculation Model and Simulation Methods

### 2.1. Model, Mesh, and Boundary Conditions

The mechanism of riblets is studied in this paper based on the standard NACA6409 airfoil, which is suitable for low-speed fans owing to its moderate thickness and large camber. The chord length *c* of the baseline airfoil is 100 mm. Each riblet-equipped airfoil model is generated by placing continuous isosceles-triangular grooves with different widths and depths. To examine the sensitivity of the airfoil suction surface (SS) to the riblet parameters, the placement location of the riblets is also considered as a variable.

**Figure 1:**
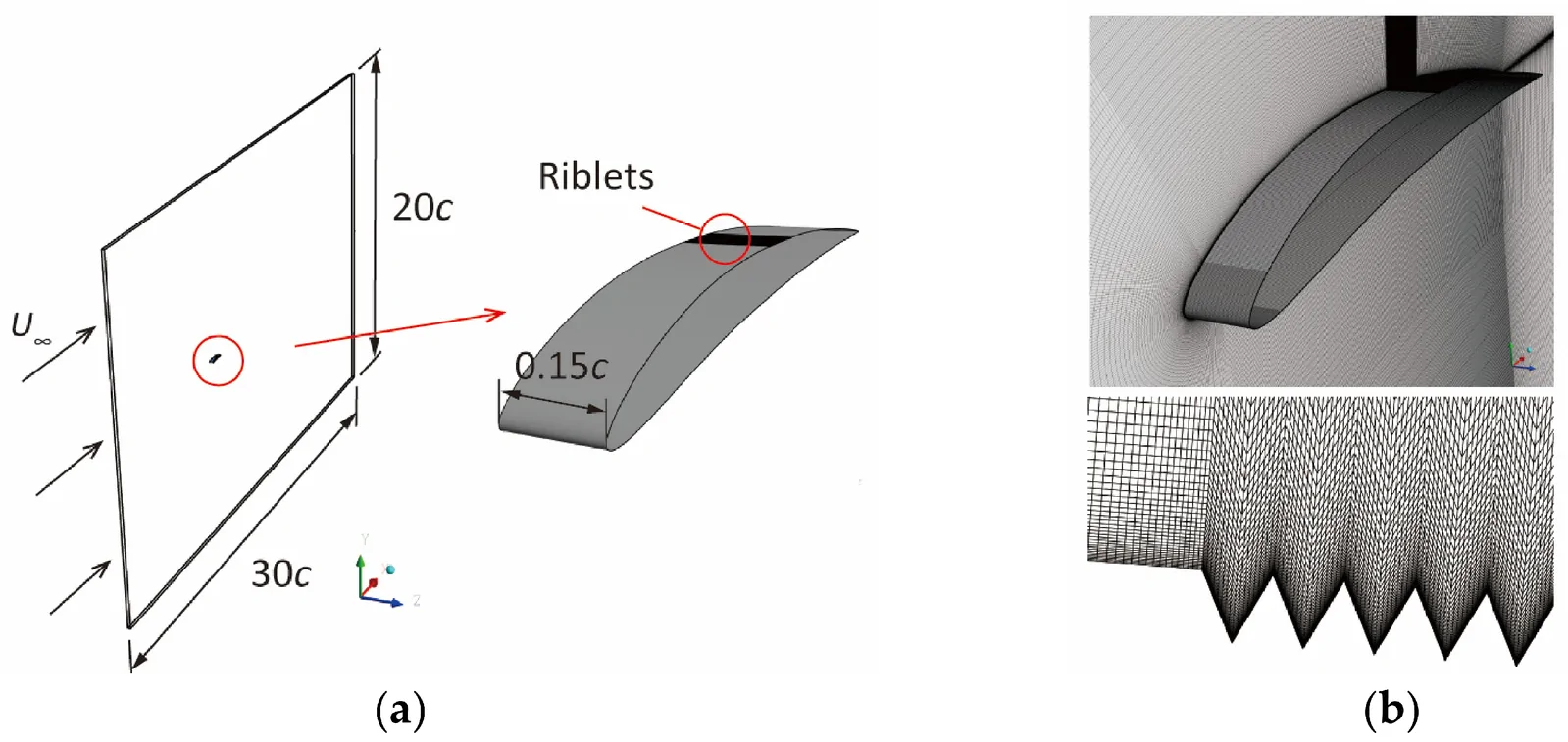
The calculation domain and mesh. (**a**) Model and calculation domain. (**b**) Mesh.

The computational mesh is automatically generated by the self-program. The computational domain has a cross-sectional size of 30*c* × 20*c* [39,40]. The upstream, upper, and lower boundaries are defined as velocity inlets, each located 10*c* away from the airfoil. The downstream boundary is a pressure outlet positioned 20*c* from the airfoil leading edge (LE). The front and back boundaries are set as periodic, coinciding with the spanwise boundaries of the airfoil. Previous high-fidelity studies have shown that spanwise extents of 0.1–0.2*c* are commonly adopted for low-angle-of-attack (AOA) transitional airfoil flows, with relatively limited effects on the mean aerodynamic characteristics and overall vortex topology [41,42]. Accordingly, *L*_z_ = 0.15*c* was adopted in the present LES considering both the representation of three-dimensional flow structures and computational cost, as shown in Figure 1.

The grid domain is divided into inner and outer grid zones. To meet the strict accuracy requirements of LES, the streamwise grid spacing ∆*x* is set to 0.2 mm and the wall-normal height of the first grid layer ∆*y* to 0.05 mm. The inner zone contains 101 nodes in the wall-normal direction, which keeps *y*^+^ < 1 and adequately resolves the near-wall flow development on the airfoil surface. The spanwise grid spacing ∆*z* is fixed at 0.5 mm to limit the total grid count while preserving accuracy. The riblet-equipped airfoils adopt the same wall-normal and spanwise node distributions. However, additional streamwise refinement is applied inside each groove, yielding local grid sizes of 0.01–0.04 mm.

### 2.2. Solving Numerical Method

In this study, large eddy simulation (LES) is employed to compute the transient flow field, and the calculations are performed using the commercial software Fluent. In LES, the large-scale turbulent structures are directly resolved, whereas the effects of unresolved small-scale motions are represented by a subgrid-scale (SGS) model [43]. The governing equations are obtained by spatially filtering the unsteady incompressible Navier–Stokes equations. The transient variable ϕ in the flow field can be decomposed into a filtered component $\bar{\phi }$ and an unresolved component $\phi^{\prime }$.

(1) $$\phi =\bar{\phi }+\phi^{\prime }$$

The filtered incompressible LES governing equations are as follows:

(2) $$\frac{\partial \bar{u_{i}}}{\partial \bar{x_{i}}}=0$$

(3) $$\rho \frac{\partial \bar{u_{i}}}{\partial t}+\rho \frac{\partial }{\partial \bar{x_{j}}}\left(\bar{u_{i}}\;\bar{u_{j}}\right)=-\frac{\partial \bar{P}}{\partial x_{i}}+\frac{\partial }{\partial x_{j}}\left(\sigma_{ij}\right)-\frac{\partial \tau_{ji}}{\partial x_{j}}$$

(4) $$\sigma_{ij}=\left[\mu_{t}\left(\frac{\partial \bar{u_{i}}}{\partial \bar{x_{j}}}+\frac{\partial \bar{u_{j}}}{\partial \bar{x_{i}}}\right)\right]-\frac{2}{3}\mu_{t}\frac{\partial \bar{u_{i}}}{\partial x_{i}}\delta_{ij}$$

where *u_i_* is the flow velocity in the *i* direction (*i*, *j* = 1, 2, 3); *p* is pressure; *t* is time; and ρ is air density. $\sigma_{ij}$ is the stress tensor caused by molecular viscosity, and *τ_ij_* is SGS stress, whose definition is:

(5) $$\tau_{ij}=\bar{u_{i}u_{j}}-\bar{u_{i}}\;\bar{u_{j}}$$

*τ_ij_* reflects the momentum transport between small-scale pulsations and solvable large-scale averages. When *u_i_* is delivered as,

(6) $$u_{i}=\bar{u_{i}}+u_{i}^{\prime }$$

*τ_ij_* can be solved as:

(7) $$\tau_{ij}=L_{ij}+C_{ij}+R_{ij}$$

(8) $$\left\{\begin{array}{c} L_{ij}=\bar{\bar{u_{i}}\;\bar{u_{j}}}-\bar{u_{i}}\;\bar{u_{j}}\\ C_{ij}=\bar{\bar{u_{i}}u_{j}^{\prime }}+\bar{u_{i}^{,}\bar{u_{j}}}\\ R_{ij}=\bar{u_{i}^{,}u_{j}^{,}} \end{array}\right.$$

Because the SGS tensor is unknown, it is necessary to construct a subgrid model to close the filtered LES governing equations. In this study, the Smagorinsky model, which is widely applied in turbulent flow research, was used. It is based on the isotropic turbulence and refers to the Reynolds average. *τ_ij_* can be expressed as:

(9) $$\tau_{ij}=\bar{u_{i}u_{j}}-\bar{u_{i}}\;\bar{u_{j}}=-2\mu_{t}\bar{S_{\mathrm{ij}}}+\frac{1}{3}\tau_{kk}\delta_{ij}$$

where $\bar{S_{ij}}$ is the rate-of-strain tensor at the resolved scale:

(10) $$\bar{S_{\mathrm{ij}}}=\frac{1}{2}(\frac{\partial \bar{u_{i}}}{\partial x_{j}}+\frac{\partial \bar{u_{j}}}{\partial x_{i}})$$

*μ_t_* is the subgrid turbulent viscosity coefficient:

(11) $$\mu_{t}={\left(C_{s}∆\right)}^{2}\left|\bar{S}\right|$$

where ∆ is the filtered length,

(12) $$\left|\bar{S}\right|\equiv \sqrt{2S_{ij}S_{ij}}$$

*C_s_* is the Smagorinsky constant.

Considering that the value of *C_s_* is different under various actual flows, the *C_s_* in this study is set as 0.1. The coupling of pressure and velocity is managed by the COUPLED algorithm and using the Bounded Central Differencing scheme to discretize the convection term.

The freestream velocity was U = 30 m/s, the chord-based Reynolds number was 2.05 × 10^5^, and the angle of attack was fixed at 2° as a representative low-incidence condition to avoid large-scale stall separation and to isolate the effects of riblet position and size. At the velocity-inlet boundaries, the ‘No Perturbations’ option in ANSYS 2022 R2 Fluent was adopted, and no synthetic velocity fluctuations were imposed on the prescribed mean inflow. No empirical transition model or prescribed transition location was employed. The laminar-to-turbulent transition developed in the resolved unsteady flow through the amplification of flow instabilities and the subsequent three-dimensional breakdown of vortical structures.

A constant time step of 6.7 × 10^−6^ s was adopted to maintain the maximum Courant–Friedrichs–Lewy number below 1. Twenty inner iterations were performed at each time step, and the normalized residuals were reduced to below 10^−6^.

The transient simulation was first advanced until the lift and drag coefficients reached statistically stationary oscillations. An additional 2600 time steps were then calculated for flow-field sampling. The final 40 lift-oscillation cycles within the sampled time series were used for statistical averaging to obtain the mean and fluctuating flow quantities used in the subsequent analysis.

### 2.3. Mesh Validation

A large number of the outer flow domain meshes hardly affects the airfoil flow simulation. To reduce the mesh number, the number of flow direction nodes from the TE to the outlet is 381, and the normal nodes’ number is 61. Table 2 shows the specific node settings of the three directions’ inner flow field.

**Figure 2:**
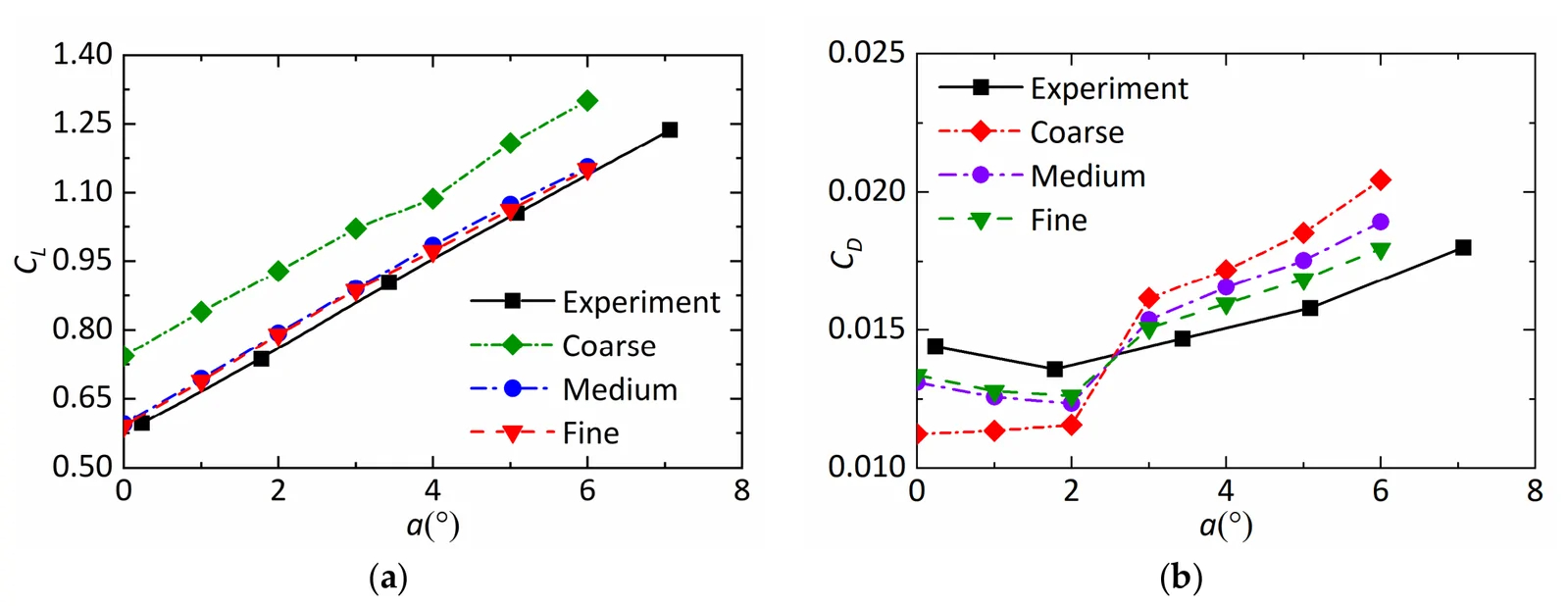
Comparison of the simulated models and experiment at 0∼6° AOA. (**a**) Lift coefficient. (**b**) Drag coefficient.

Figure 2 compares the simulated averaged lift and drag coefficients of partial mesh models with the experimental data available in Ref. [44] under 0–6° AOA. As shown in Figure 2, the coarse mesh exhibits noticeable deviations, whereas the medium and fine meshes give similar aerodynamic coefficients. At 2° AOA, the difference in *C_D_* between the medium and fine meshes is approximately 2.1%, while the variation in *C_L_* is also small. The numerical results reproduce the overall trends of both *C_L_* and *C_D_* observed in the experiments. Therefore, considering the calculation accuracy and computing resources allocation, the grid number of the internal domain is 1671 × 121 × 31, while the external domain’s is 2431 × 61 × 31. Thus, the total grid number of the original model is about 10.4 million.

## 3. Results and Discussion

### 3.1. Definition of Airfoil Suction Surface and Riblets Design

**Figure 3:**
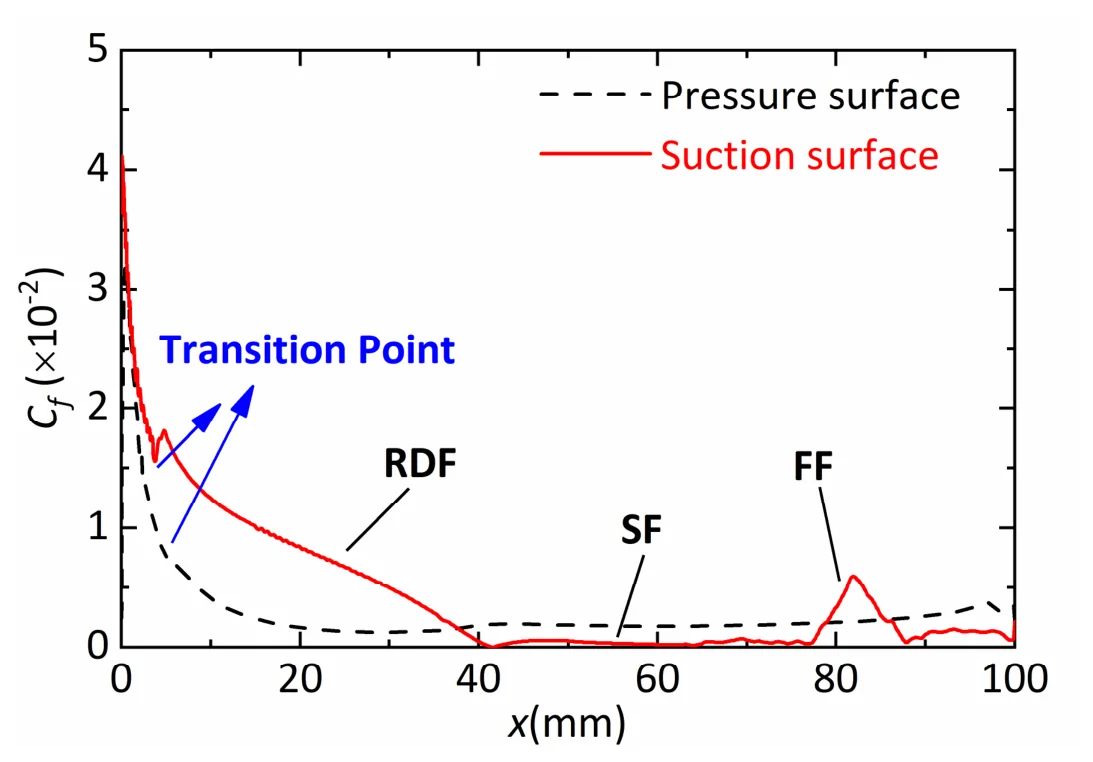
Friction coefficient distribution of the smooth baseline airfoil at 2° AOA.

The drag reduction mechanism of riblets is primarily associated with the near-wall dynamics of turbulent boundary layers. Therefore, the airfoil flow morphology over the airfoil suction surface was first characterized before determining the riblet locations. The transition of the airfoil flow from laminar to turbulent flow is shown in Figure 3 by the friction coefficient distribution of the airfoil surface. Under the present condition, the transition location on the SS is approximately *x*/*c* = 0.038. Based on the turbulent region, the airfoil SS can be divided into three parts: the rapidly decreasing region (RDR), the stable region (SR), and the fluctuating region (FR).

To investigate the combined effects of chordwise location and riblet size on airfoil performance, riblets with equal height and spacing (*h*=*s*), where *h* denotes the riblet height and *s* denotes the riblet spacing, were arranged over 0.1–0.2*c*, 0.25–0.35*c*, 0.5–0.6*c*, and 0.7–0.8*c* of the airfoil SS, respectively. The geometric dimensions were additionally expressed in viscous wall units as:

(13) $$h^{+}=\frac{hu_{\tau }}{\nu },\;\;s^{+}=\frac{su_{\tau }}{\nu }$$

where $u_{\tau }$ is the friction velocity. Because the wall shear stress varies along the suction surface, a representative friction velocity was evaluated separately for each riblet region from the spatially and temporally averaged skin-friction coefficient of the corresponding region on the smooth baseline airfoil:

(14) $$u_{\tau }=U_{\infty }\sqrt{\frac{\bar{C_{f}}}{2}}$$

The corresponding dimensional and viscous-scaled parameters of all riblet configurations are summarized in Table 3.

### 3.2. Lift and Drag Coefficients

**Figure 4:**
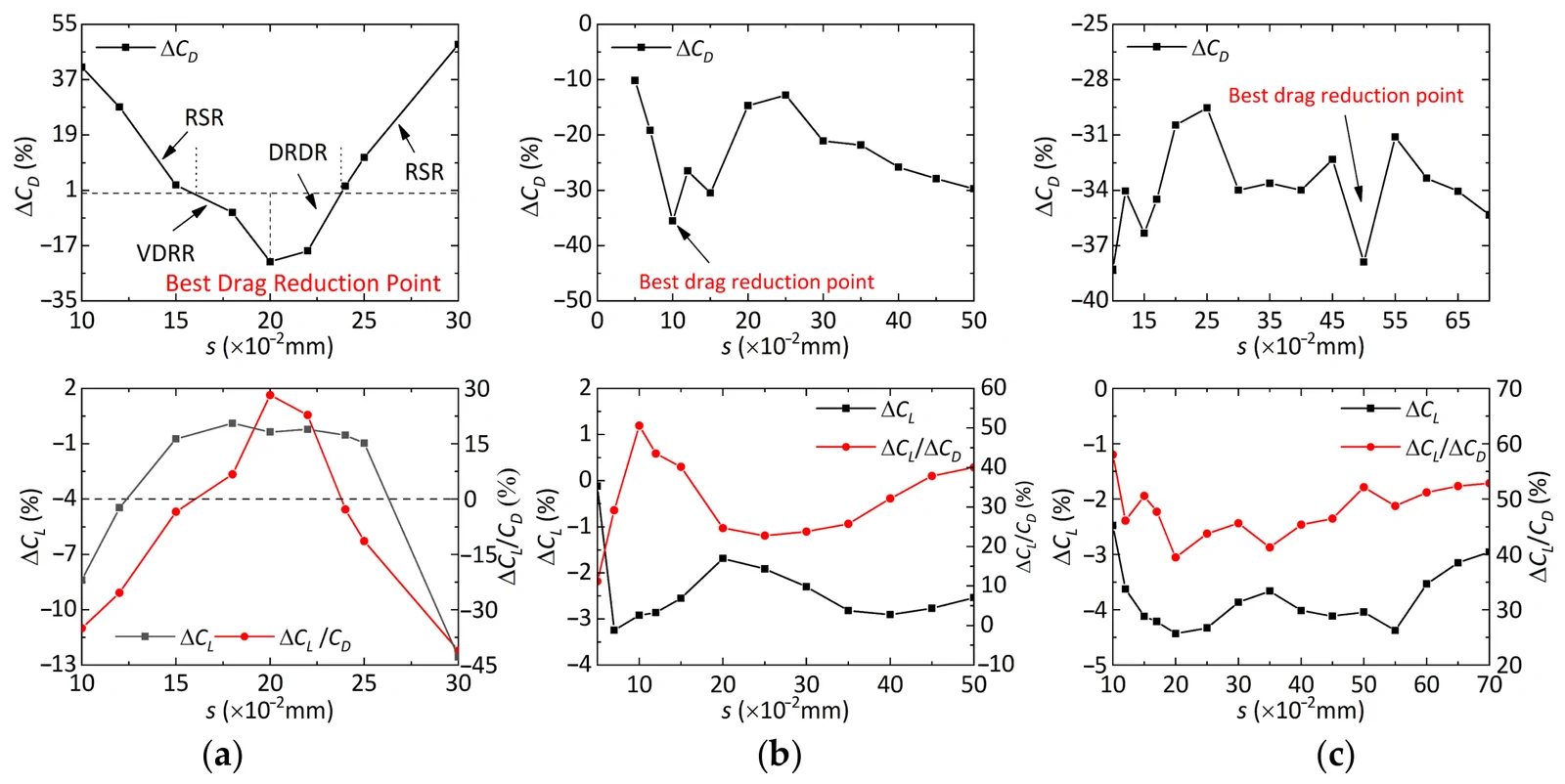
Relative changes in the aerodynamic coefficients for different riblet configurations. (**a**) Riblets located at 0.5–0.6*c*. (**b**) Riblets located at 0.1–0.2*c*. (**c**) Riblets located at 0.25–0.35*c*.

Figure 4 compares the relative changes in the averaged aerodynamic coefficients for the different riblet configurations. A negative value of $∆C_{D}$% indicates a reduction in the mean drag coefficient relative to the smooth baseline airfoil. As shown in Figure 4a, for the riblets located at 0.5–0.6*c*, the magnitude of drag reduction initially increases with the riblet size, reaches an optimum, and subsequently deteriorates. The variation on the large-size side of the optimum exhibits the characteristics of the classical riblet drag reduction behavior. That is, they can be divided into a viscous drag reduction region (VDRR), best drag reduction point, drag reduction deterioration region (DRDR), and rough surface region (RSR). In contrast, an additional drag-increase regime is observed for the smallest riblet sizes before the VDRR. The maximum drag reduction of 22.18% occurs at *h* = *s* = 0.2 mm, corresponding to *h^+^* = *s^+^* = 5.33. When the riblet size exceeds approximately *h* = *s* = 0.24 mm (*h^+^* = *s^+^* ≈ 6.39), the airfoil drag coefficient increases rapidly. However, the riblets at 0.5–0.6*c* cannot raise the airfoil lift coefficient. Thus, the average lift–drag ratio of riblet airfoil also increases first and then decreases, and the maximum increase is 28.18% at *h* = 0.2 mm. However, when *h* = 0.3 mm, the value decreased by 41.1% compared with the original. These results show that the effect of riblets depends on both their size and chordwise location. Since the wall shear stress changes along the airfoil surface, the same riblet size corresponds to different *h^+^* and *s^+^* values at different locations, leading to different drag reduction performance.

**Figure 5:**
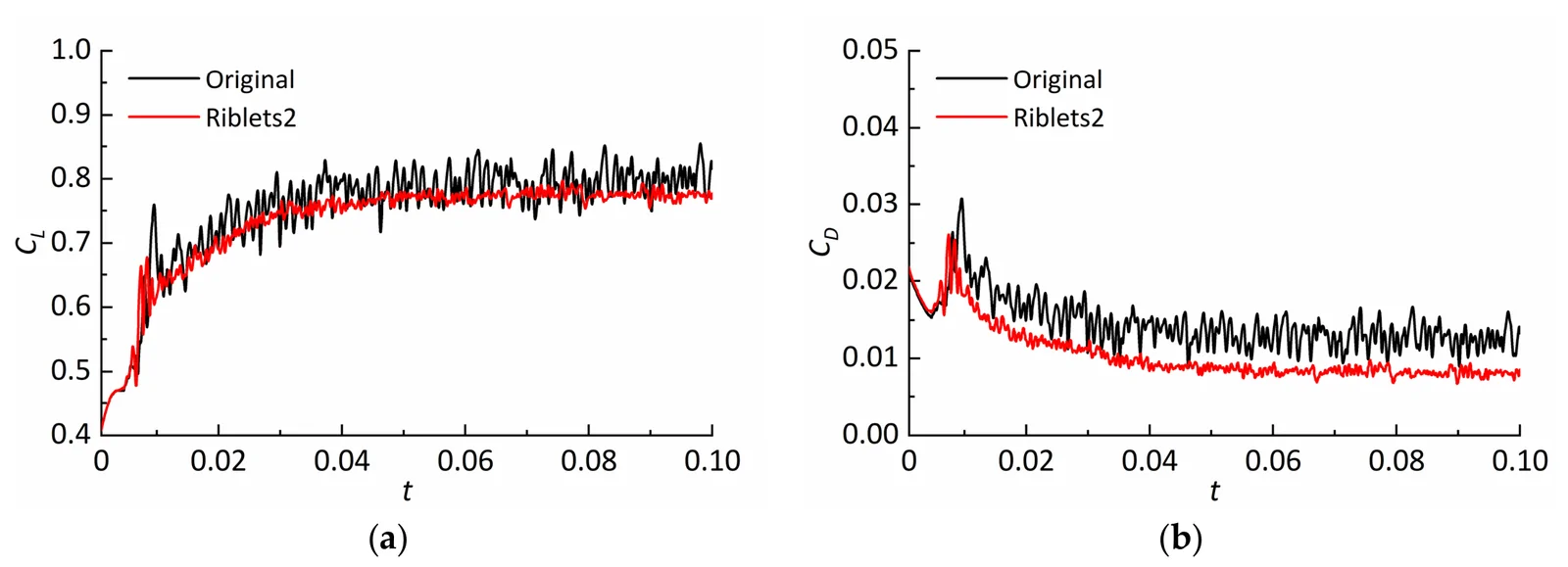
The transient *C_L_* and *C_D_* of the original and the drag reduction scheme. (**a**) Lift coefficient. (**b**) Drag coefficient.

Figure 5 compares the transient *C_L_* and *C_D_* of the original and the drag reduction scheme with riblets of 0.1 mm at 0.1–0.2*c*. After the initial transient stage, both *C_L_* and *C_D_* exhibit smaller fluctuations for the ribleted airfoil. The riblets modify the near-wall momentum exchange in the 0.1–0.2*c* region and weaken the development of large-scale unsteady vortical structures downstream. As a result, the associated pressure and wall-shear fluctuations are reduced, leading to smaller oscillations in the aerodynamic coefficients. This behavior is consistent with the vortex-structure analysis presented below.

### 3.3. Drag Reduction Mechanism

To investigate how riblet arrangement affects airfoil flow, the flow fields of the schemes in which riblets reduced drag in the SR and RDR and increased drag in the FR are compared with the original. Table 4 lists the configuration of these specific schemes, they are: the riblets of 0.2 mm in 0.5–0.6*c*, 0.1 mm in 0.1–0.2*c* and 0.1 mm in 0.7–0.8*c* (referred to as Riblets1, Riblets2, Riblets3, respectively).

**Figure 6:**
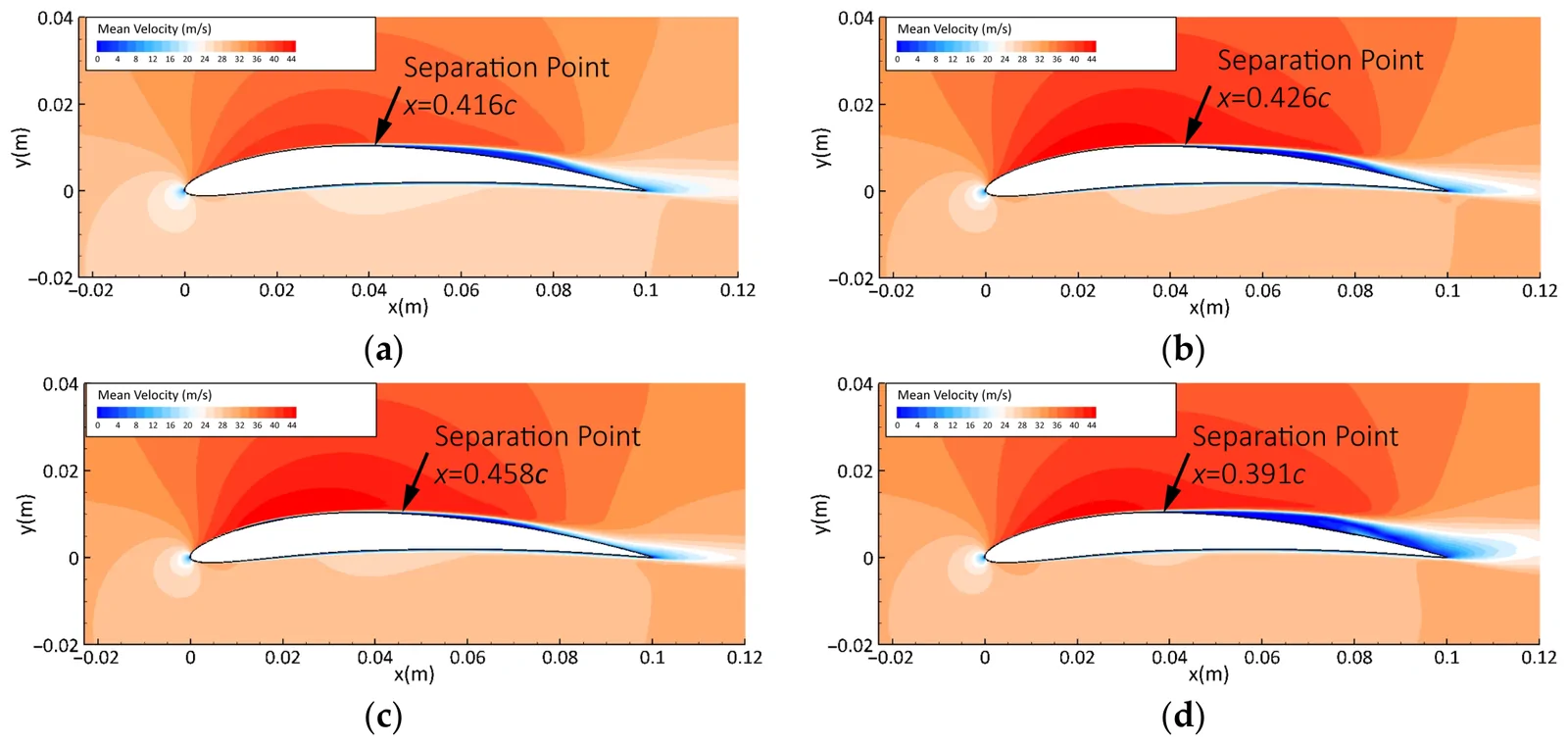
The average velocity contours of the airfoils. (**a**) Original. (**b**) Riblets1. (**c**) Riblets2. (**d**) Riblets3.

The time-averaged velocity contours of the original and riblets schemes are compared in Figure 6. The low-speed fluid area at the Riblets1 and Riblets2 schemes’ SS is smaller than that of the original. Their separations at the trailing edge are suppressed, and the separation point shifts backward. The flow of Riblets1 separates at 0.426*c*, which is 2.38% later than the original’s 0.416*c*. For Riblets2, it not only has a 9.59% later separation point but also presents a thin separation bubble on the SS. It can be seen that the mainstream flow attaches quickly to the airfoil wall. Therefore, the riblets arranged in the airfoil’s RDR have the best flow control effect.

However, riblets in the airfoil’s FR promote flow separation. Riblets3’s separation bubble size increases significantly, and the separation point moves forward. The inverse velocity gradient inside the separation vortex is also raised. Meanwhile, the high-speed fluid area on the airfoil SS is smaller than the other riblets schemes, indicating that these riblets reduce the flow velocity on the airfoil.

**Figure 7:**
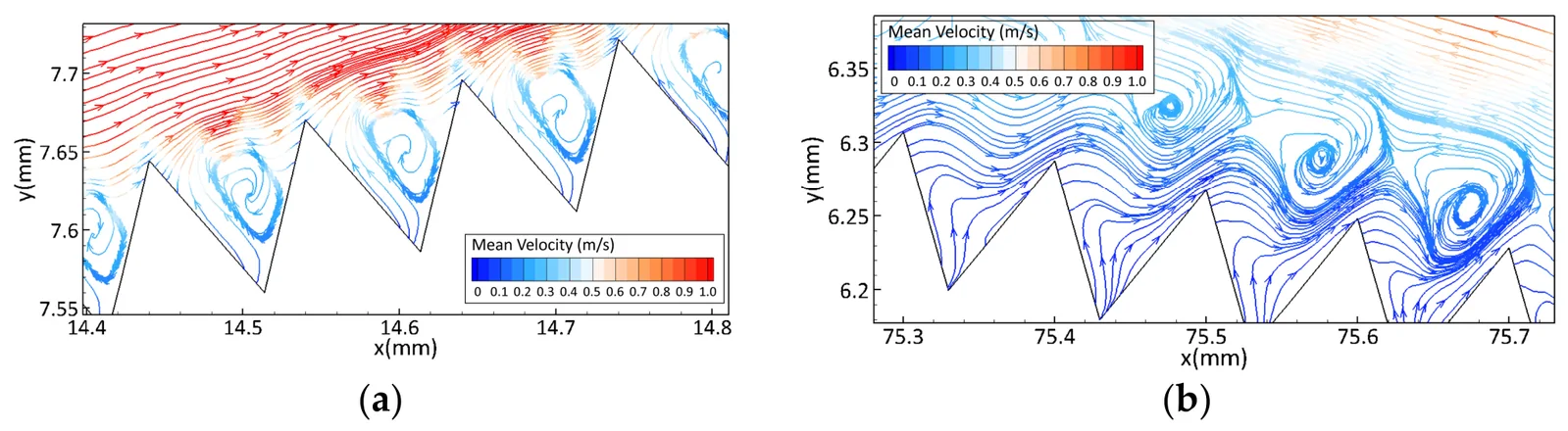
Local streamline distributions within the riblet grooves under different flow conditions. (**a**) Riblets2 in the attached-flow region at 0.1–0.2*c*. (**b**) Riblets3 in the separated-flow region at 0.7–0.8*c*.

Figure 7 compares the streamline patterns within the riblet grooves under two different near-wall flow conditions. Vortexes for which rotation directions are the same with the main flow direction are formed in the Riblets2 grooves. These vortexes act as rolling bearings, reducing the friction between the main flow and the airfoil wall.

In contrast, Riblets3 are located in a separated-flow region at 0.7–0.8*c*, where the grooves are largely occupied by reverse flow. Thus, the vortexes are formed above the riblets and rotate in the same direction as the reverse flow, resulting in the reverse flow moving further upstream and the increased airfoil drag. Consequently, whether riblets enhance or reduce drag is determined by whether the rotation direction of the groove’s vortex is congruent with the main flow.

**Figure 8:**
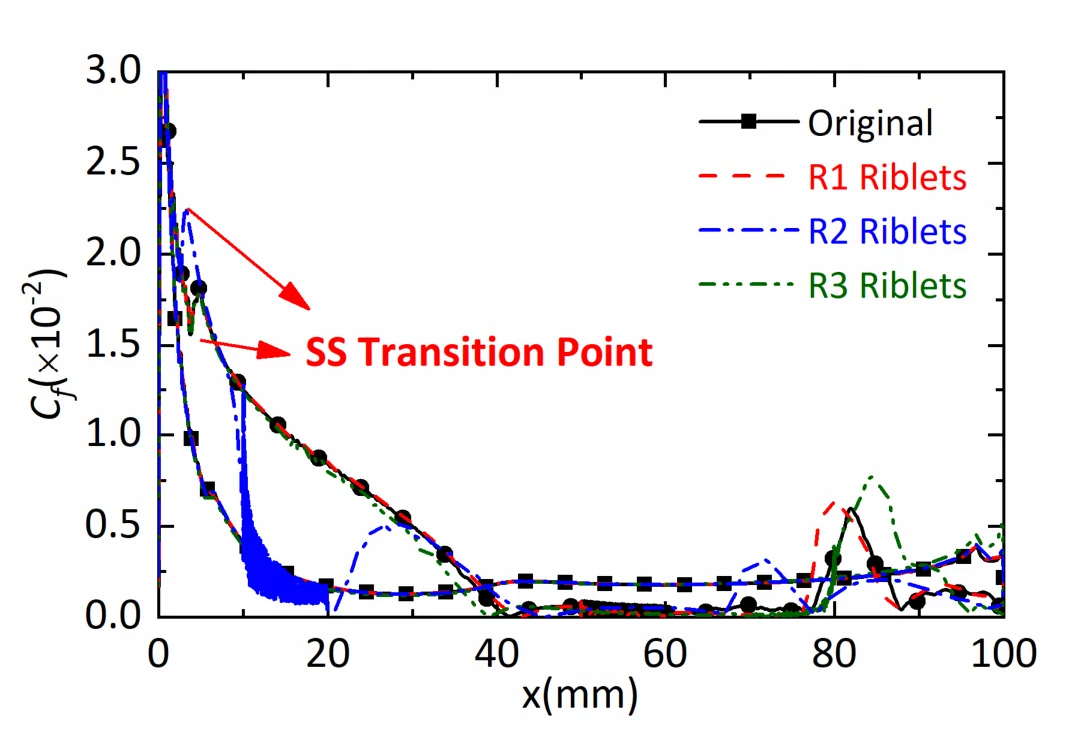
The average friction coefficients of the airfoils.

Frictional drag dominates airfoil viscous drag. The average friction coefficients of the schemes are shown in Figure 8. On one hand, riblets affect the friction coefficient of the airfoil’s pressure surface very little. On the other hand, both the friction coefficients of Riblets1 and Riblets2 where the riblets are arranged are reduced. This decrease is brought on by the small velocity gradient inside the groove, which lessens the wall friction drag. Because Riblets2 is positioned where the airflow is accelerating, the friction coefficient drops more and the SS’s transition point moves forward. However, Riblets3 raises the friction coefficient from 0.83*c* to 0.94*c* considerably. Thus, a partial reason for the Riblets3 drag growth is the increased friction at the airfoil TE.

**Figure 9:**
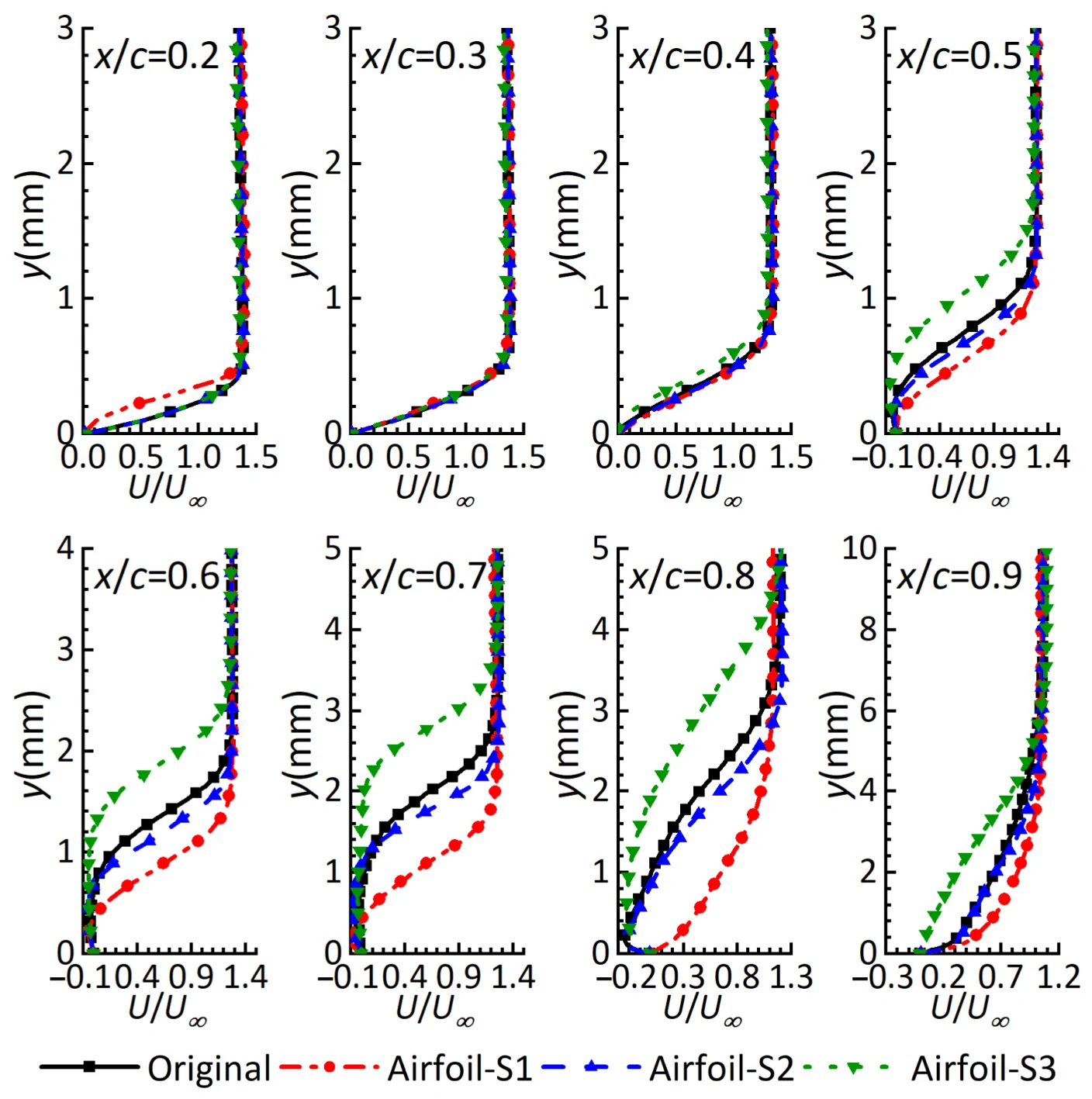
The average velocity distribution in the boundary layers of the airfoils.

Figure 9 illustrates the velocity distribution of the schemes’ boundary layers. To acquire the velocity distribution at various locations on the airfoil SS, monitoring points are placed every 10% chord length. At *x*/*c* = 0.2, only Riblets2 has a smaller velocity distribution than the other three airfoils because of its riblet arrangement. Meanwhile, the other two riblet airfoils have the same velocity distribution as the original at *x*/*c* = 0.2–0.3. The inverse velocity gradient appears near the original’s wall at 0.4*c*. As the monitoring point moves backward from 0.4*c* to 0.7*c*, the normal height of the inverse velocity rises, and the airfoil boundary layer separation intensifies. The inverse velocity gradient weakens after reaching a maximum at 0.8*c* and decreases to zero at 0.9*c*. The separation vortex on the SS disappears, and the streamline of the main flow reattaches to the airfoil wall.

### 3.4. Vortex Analysis Based on Q-Criterion

The Q-criteria identification method based on the second invariant of the velocity gradient tensor is a widely used method for vortex structures. Its formula is as follows:

(15) $$Q=\frac{1}{2}\left(\Omega_{ij}\Omega_{ij}-S_{ij}S_{ij}\right)$$

where Ω refers to vorticity, which can be defined as:

(16) $$\Omega_{ij}=\frac{1}{2}(\frac{\partial u_{i}}{\partial x_{j}}-\frac{\partial u_{j}}{\partial x_{i}})$$

*x_i_* is the direction, *u_i_* is the velocity component in the *x_i_* direction; and

*S_ij_* is the strain rate tensor, whose expression is:

(17) $$S_{ij}=\frac{1}{2}(\frac{\partial u_{i}}{\partial x_{j}}+\frac{\partial u_{j}}{\partial x_{i}})$$

**Figure 10:**
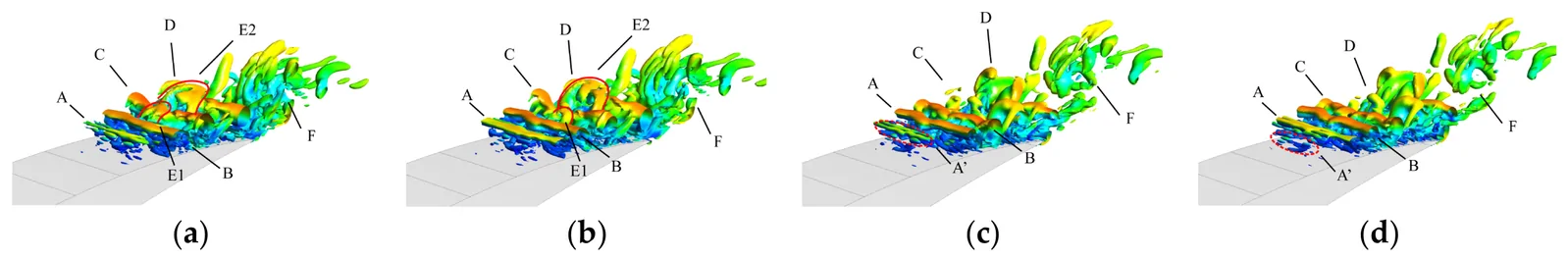
Formation and development of the original vortex structure. (**a**) *T* = 48.843 s. (**b**) *T* = 48.8842 s. (**c**) *T* = 49.0038 s. (**d**) *T* = 49.044 s.

Figure 10 shows the vortex structure of the original based on the instantaneous velocity *Q* iso-surface at the dimensionless time T=48.843–49.0842 and Q=0.05. The dimensionless time *T* is defined as:

(18) $$T=t\frac{U_{\infty }}{c}$$

where $U_{\infty }$ is the main flow velocity, and *c* is the characteristic length.

At the middle of the SS, the fluid forms a lower velocity vortex near the wall. When moving downstream, the vortex is induced to rise gradually. After entering the high-velocity zone, it is continuously distorted and fragmented into small, slender vortexes as a result of the sharp increase in internal vorticity.

In Figure 10a, vortex A is in the early stage of formation, while vortex B and C are completely developed. However, the induced velocity of the E1 vortex filament pair is greater than that of other positions. Its head lifts faster than other vortex filament pairs in vortex C. Therefore, the pair of vortex filament in vortex D at the same position forms an Ω-shaped structure at the E2 point. When the vortex moves to point F, it is pulled and broken by the high-speed fluid above, forming numerous slender vortexes.

At the moment of Figure 10b, vortex A is growing gradually as it moves downstream, and a small quantity of low-speed vortex begins to appear in front of it. Influenced by the induced velocity, the outer vortex filament pair of vortex C exits the wall faster and develops to the upper high-speed region, gradually mixing with vortex D.

**Figure 11:**
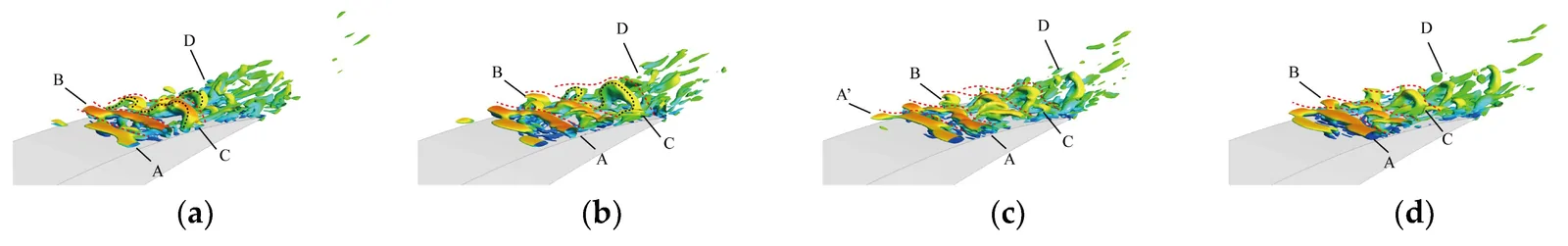
Vortex development of the airfoil with Riblets2. (**a**) *T* = 48.843 s. (**b**) *T* = 49.044 s. (**c**) *T* = 49.1244 s. (**d**) *T* = 49.2048 s.

Figure 11 shows the vortex development of Riblets2 The scheme consists primarily of four features: vortex A, B, C, and D. Each vortex is close to the airfoil surface at all times and only rises to a certain height when it is about to fall off from the airfoil TE. Compared with the original, the quantity, strength, and lifting height of the vortexes are significantly reduced on the riblet airfoils. It can be seen that the main reason Riblets2 is effective in reducing drag is because it inhibits the vortexes’ formation and their development in the flow direction and normal direction.

### 3.5. Application of Drag Reduction Riblets to a Backward Centrifugal Fan

Based on the quasi-three-dimensional airfoil results, a riblet configuration was selected for application to the backward centrifugal fan and subsequently evaluated experimentally.

#### 3.5.1. Riblet Selection

The quasi-three-dimensional LES results show that riblets located at 0.1–0.2*c* of the NACA6409 suction surface provide relatively strong drag reduction performance over a range of riblet sizes. This region was therefore selected for the subsequent fan application.

To evaluate the applicability of the fixed-airfoil results to the rotating blade, three representative blade sections at *z* = 24, 48, and 72 mm were examined. The estimated local chord Reynolds numbers of 2.5 × 10^5^ to 3.7 × 10^5^, comparable in scale to the reference value Re = 2.05 × 10^5^, was used in the quasi-three-dimensional airfoil simulations. The spatially averaged C*_f_* values over the riblet region range from 3.37 × 10^−3^ to 4.01 × 10^−3^, indicating similar near-wall shear conditions among the selected sections. These local flow characteristics indicate that the fixed-airfoil results can serve as a practical reference for the subsequent rotating-blade study.

For the 0.1–0.2*c* region, the maximum drag reduction predicted by the airfoil LES is approximately 35% at *h* = *s* = 0.1 mm, while the *h* = *s* = 0.5 mm configuration still achieves a drag reduction of 29.7% while offering better manufacturability for 3D printing. Considering both drag reduction performance and manufacturing feasibility, riblets with *h* = *s* = 0.5 mm were therefore selected and arranged over 0.1–0.2*c* of the blade suction surface.

#### 3.5.2. Experimental Design and Test Procedure

**Figure 12:**
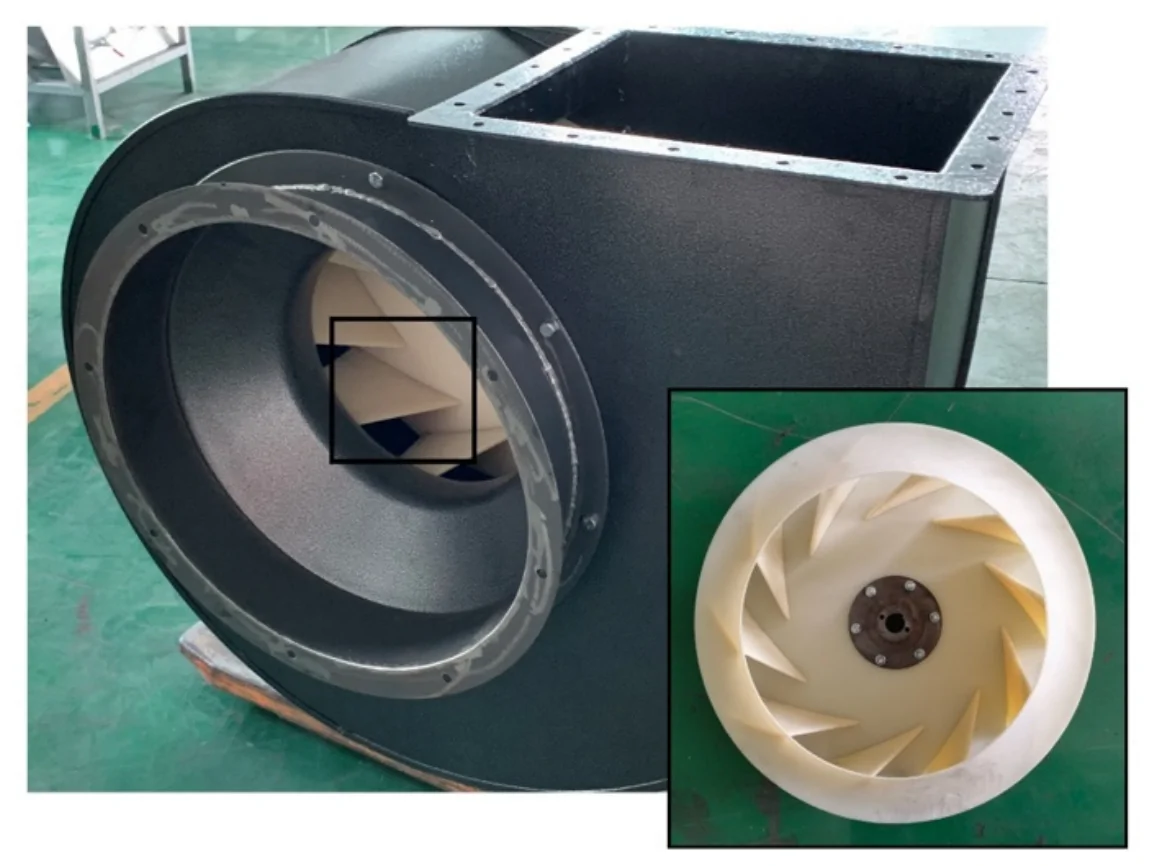
The backward centrifugal fan with the blade section shape of NACA6409 airfoil.

The configuration of the backward centrifugal fan with NACA6409 blade sections is shown in Figure 12, and the main geometric and operating parameters of the impeller are listed in Table 5.

**Figure 13:**
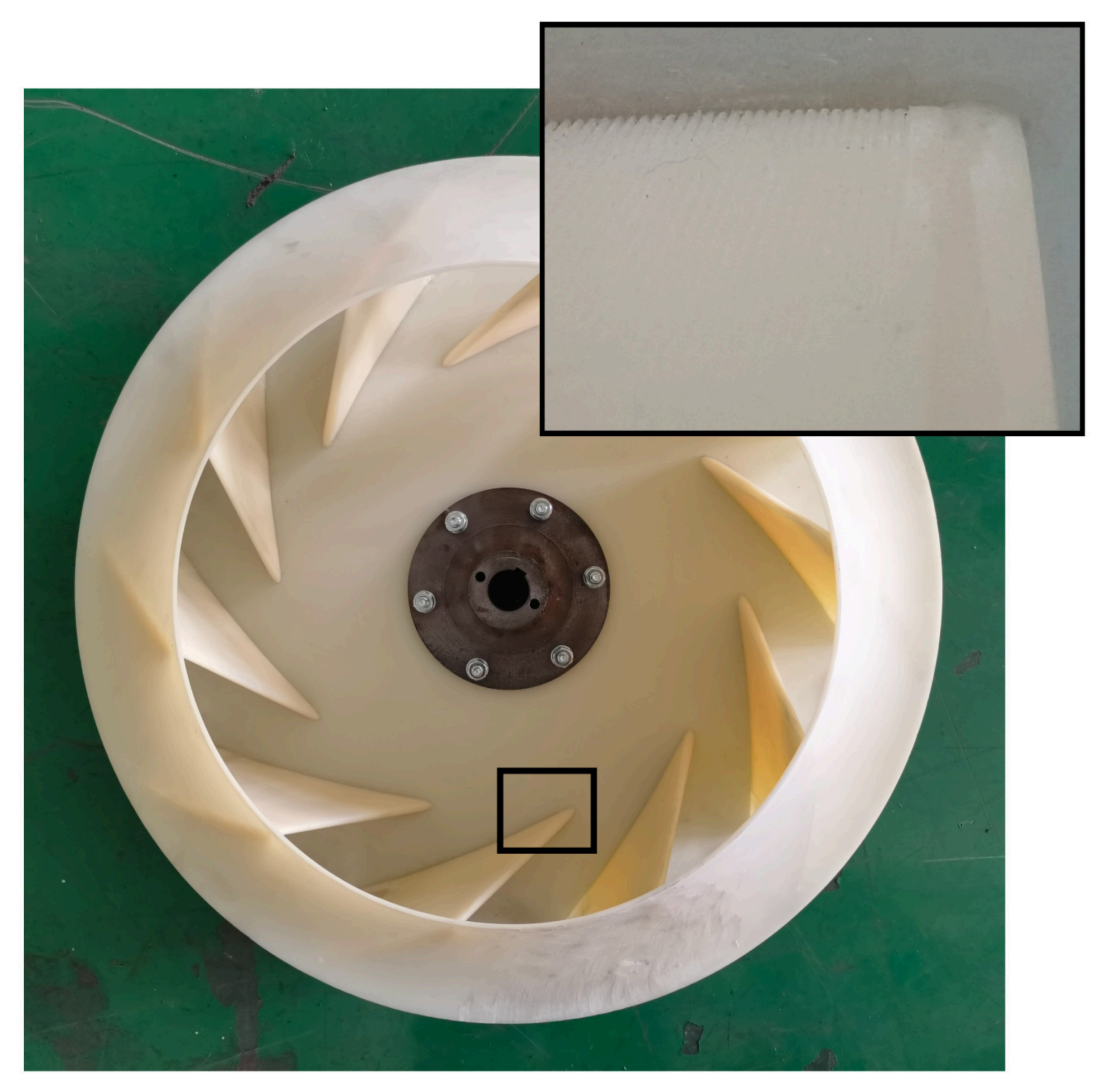
The impeller with a ribleted blade.

Two impellers with identical baseline geometry were prepared for comparison: a smooth impeller and a ribleted impeller. Both impellers were manufactured by a professional third-party company using 3D printing based on the CAD models developed in this study. The ribleted impeller followed the configuration selected above, and photographs of the fabricated impeller and local riblet details are shown in Figure 13.

**Figure 14:**
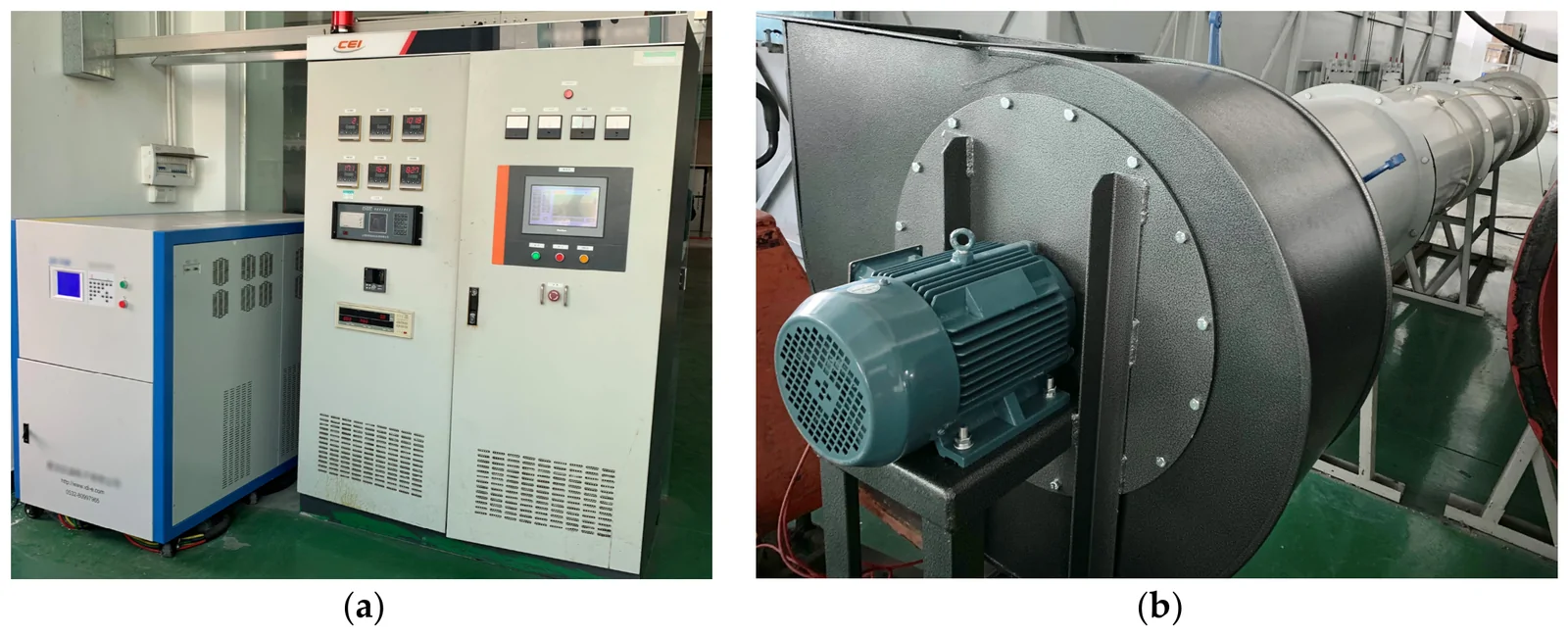
Experimental setup for the centrifugal-fan performance test. (**a**) Measurement and control system; (**b**) fan test bench.

The aerodynamic performance tests were conducted on a C-type fan test bench in accordance with GB/T 1236-2017. The test system employed a ducted inlet and free outlet, as shown in Figure 14. Photographs of the test fan assembly and complete experimental setup are provided in Figure S1 and Figure S2, respectively. The static pressure at the specific measured section of the induced duct was sensed by averaging four pressure taps located 90° apart around the measuring duct. The volume flow rate was then calculated from the static pressure obtained by the pressure taps, the air density, and the cross-sectional area of the measuring duct. The pressure taps had a measurement range of −1000 to 1000 Pa and an accuracy of ±1.5 Pa, which is significantly smaller than the pressure range of the fan measured in the present study. The rotational speed was measured using an optical rotational-speed sensor with a range of 0–5000 rpm and an accuracy of ±5 rpm. Considering that the rotational speed of the tested fan is 1450 rpm, this rotational-speed uncertainty can be considered negligible. In addition, the ambient temperature of the experiment was maintained within 20 ± 5 °C, and the relative humidity was below 85%.

The fan operation condition was adjusted by varying the inlet area. After each adjustment, the fan was operated for at least 3 min to reach a stable condition before data acquisition. During the test, the acquisition program processed the instantaneous flow rate and pressure data at 5 s intervals. The measurements were recorded continuously for 2 min, and the time-averaged values over this period were used to determine the aerodynamic performance at each operating point. According to the test bench instructions and the testing standards it follows, this testing process can ensure the uncertainty of the measured aerodynamic performance to be less than 1%.

#### 3.5.3. Experimental Results and Discussion

**Figure 15:**
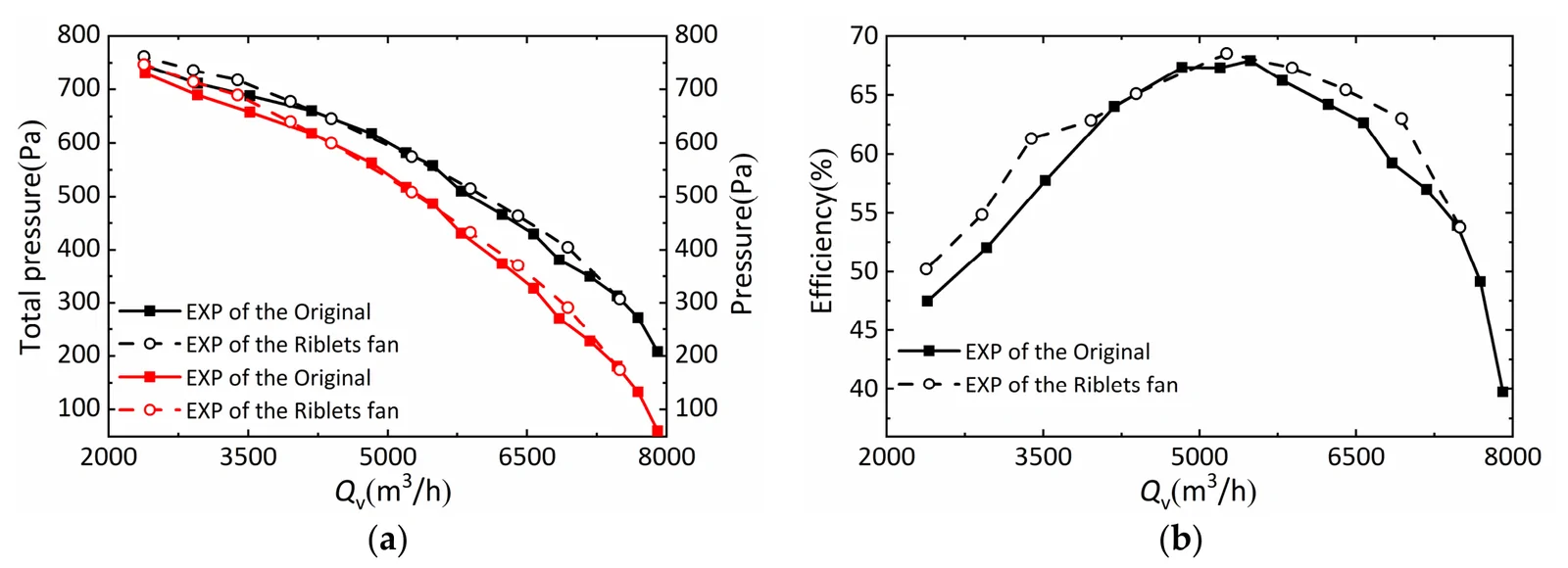
Experimental aerodynamic performance of the smooth and ribleted fans. (**a**) Total and static pressure. (**b**) Efficiency.

Figure 15 compares the measured aerodynamic performance of the smooth and ribleted fans over the investigated flow rate range. The best efficiency point (BEP) of the smooth fan occurs at approximately *Q*_v,BEP_ = 5500 m^3^/h. At low flow rates, the ribleted fan generally produces higher pressure than the smooth fan. The maximum increases in total and static pressure are approximately 4.7% and 4.2%, respectively, at 0.63 Q_v,BEP_. As the flow rate approaches Q_v,BEP_, the pressure difference between the two fans gradually decreases. Above the BEP, a slight pressure improvement is maintained over part of the operating range.

A similar trend is observed for efficiency. The ribleted fan exhibits a more evident improvement under low-flow conditions, with a maximum efficiency increase of approximately 4.5% at 0.63 *Q*_v,BEP_. The difference becomes small as the flow rate increases. These results indicate that the effect of the riblets is strongly dependent on the operating condition, with the main performance benefit occurring in the low-flow region.

## 4. Conclusions

In this study, riblets oriented perpendicular to the flow direction were applied to the suction surface of an NACA6409 airfoil. Large eddy simulation (LES) was employed to numerically analyze how the riblet parameters, evaluated through the friction coefficient distribution, influence the flow characteristics. Riblets that exhibited effective drag reduction were subsequently applied to a backward centrifugal fan. The main conclusions are as follows:

(1) The sensitivity of the airfoil suction surface to riblet placement was characterized. The rapidly decreasing friction region (RDR) offers considerable flexibility in groove size. Within 0.1–0.2 chord lengths, drag reduction is achieved for groove sizes from 0.05 mm to 0.5 mm, and within 0.25–0.35 chord lengths, for sizes from 0.1 mm to 0.7 mm. The maximum drag reductions of 35.54% and 37.89% were obtained with 0.1 mm riblets at 0.1–0.2 chord lengths and 0.5 mm riblets at 0.25–0.35 chord lengths, respectively. In the stable friction region (SR), the aerodynamic characteristics are highly sensitive to groove size, and drag is reduced only when the size lies between 0.16 mm and 0.237 mm. In the fluctuating friction region (FR), riblets of any size degrade the airfoil performance. Therefore, positioning riblets in the RDR allows for the effective size to be chosen from a wide range.
(2) The primary drag reduction mechanism of riblets perpendicular to the flow is as follows: a vortex rotating in the same direction as the main flow forms inside the groove and acts like a rolling bearing, thereby reducing the velocity gradient near the wall and the viscous drag. Moreover, riblets of an appropriate size effectively suppress vortex separation by reducing the vortex strength, lift-off height, and shedding frequency. This leads to lower friction drag and weaker turbulent dissipation in the boundary layer, which thins the boundary layer and markedly improves the near-wall velocity gradient. In contrast, riblets of an unsuitable size increase the boundary layer thickness and aggravate the formation and development of separation vortices.
(3) It is advisable to place riblets of a suitable size near the blade leading edge, where the friction coefficient decreases sharply. Experimental results confirm that 0.5 mm riblets located over 0.1–0.2 chord lengths from the leading edge increase the fan efficiency by about 4.5% and the total pressure by about 4.2% under low-flow conditions, respectively. The fan performance is also improved under most operating conditions.

## Figures and Tables

**Table 1:** Drag reduction effect of different riblets in partial research.

Author	Layout Object	Section Shape	Flow Condition	Maximum Drag Reduction
Kim et al. [22]	flat	V-type	Re*_L_* = 1.2 × 10^7^	3%
Hou et al. [23]	flat	trapezoid	Re*_H_* = 4780	6.1%
Fu et al. [24]	flat	concave	Re = 12,200–24,200	6.5%
Hijazi et al. [25]	airfoil	hybrid riblet	Re = 30,000	8.36%
Yang et al. [26]	airfoil	sawtooth	Re = 100,000	15.04%
Wu et al. [20]	airfoil	sawtooth	Re = 2.88 × 10^6^	9.65%
Domel et al. [21]	airfoil	shark denticle	Re*_c_* = 40,000	28%
Li et al. [27]	airfoil	sawtooth	U = 60 m/s	39.8%

**Table 2:** The three directions’ nodes of the inner flow field.

	Flow Direction	Normal Direction	Spanwise Direction
Coarse	1241	81	21
Medium	1671	121	31
Fine	1951	151	41

**Table 3:** Geometric and viscous-scaled dimensions of the riblet configurations.

Region	Case	*h*	*s*	*h^+^*	*s^+^*
0.1–0.2*c*	A1	0.05	0.05	7.33	7.33
	A2	0.10	0.10	14.66	14.66
	A3	0.15	0.15	22.00	22.00
	A4	0.30	0.30	43.99	43.99
	A5	0.40	0.40	58.65	58.65
	A6	0.50	0.50	73.32	73.32
0.25–0.35*c*	B1	0.10	0.10	10.17	10.17
	B2	0.15	0.15	15.25	15.25
	B3	0.20	0.20	20.34	20.34
	B4	0.30	0.30	30.51	30.51
	B5	0.40	0.40	40.68	40.68
	B6	0.50	0.50	50.85	50.85
	B7	0.60	0.60	61.02	61.02
	B8	0.70	0.70	71.19	71.19
0.5–0.6*c*	C1	0.10	0.10	2.66	2.66
	C2	0.12	0.12	3.20	3.20
	C3	0.15	0.15	4.00	4.00
	C4	0.18	0.18	4.79	4.79
	C5	0.20	0.20	5.33	5.33
	C6	0.22	0.22	5.86	5.86
	C7	0.24	0.24	6.39	6.39
	C8	0.25	0.25	6.66	6.66
	C9	0.30	0.30	7.99	7.99
0.7–0.8*c*	D1	0.10	0.10	4.13	4.13
	D2	0.30	0.30	12.40	12.40

**Table 4:** Riblets schemes.

	*h*	*s*	Location
Riblets1	0.2 mm	0.2 mm	0.5–0.6*c*
Riblets2	0.1 mm	0.1 mm	0.1–0.2*c*
Riblets3	0.1 mm	0.1 mm	0.7–0.8*c*

**Table 5:** Impeller parameters.

Parameter	Value
Outer radius	500 mm
Blade number	10
Blade angle	48°
Impeller axial length	164 mm
Impeller outlet width	123.5 mm
Design rotation speed	1450 rpm

## Data Availability

The data are available from the corresponding author on reasonable request.

## Supplementary Materials

The following supporting information can be downloaded at: https://www.mdpi.com/article/10.3390/biomimetics11100694/s1, Figure S1: The test fan with a 3D-printed centrifugal impeller; Figure S2: The experimental platform with the test fan installed.

## Abbreviations

AOA	Angle of attack
BEP	Best efficiency point
DRDR	Drag reduction deterioration region
FR	Fluctuating region
LE	Leading edge
LES	Large eddy simulation
RDR	Rapidly decreasing region
RSR	Rough surface region
SGS	Subgrid scale
SR	Stable region
SS	Suction surface
TE	Trailing edge
VDRR	Viscous drag reduction region
*x*	Airfoil chordwise location
*y*	Airfoil normal location
*z*	Airfoil spanwise location
*c*	Airfoil chord length
*C_f_*	Friction coefficient
*C_L_*	Lift coefficient
*C_D_*	Drag coefficient
*L*_z_	Airfoil spanwise width
*h*	Riblet height
*h* ^+^	Dimensionless riblet height
*s*	Riblet spacing
*s* ^+^	Dimensionless riblet spacing
Q	Q-criterion vorticity
*Q*_v_	Volume flow rate
*t*	Time
*T*	Dimensionless time
