# Supplementary material for: Drag Reduction Mechanism of Bionic Riblets and Their Application on Centrifugal Fan Blades
Source: Biomimetics (Basel). 2026 Sep 29;11(10):694. doi: 10.3390/biomimetics11100694 (PMC13702765; doi:10.3390/biomimetics11100694)
Supplement: Supplementary file 1 [file biomimetics-11-00694-s001.zip › biomimetics-4499316-supplementary.pdf]

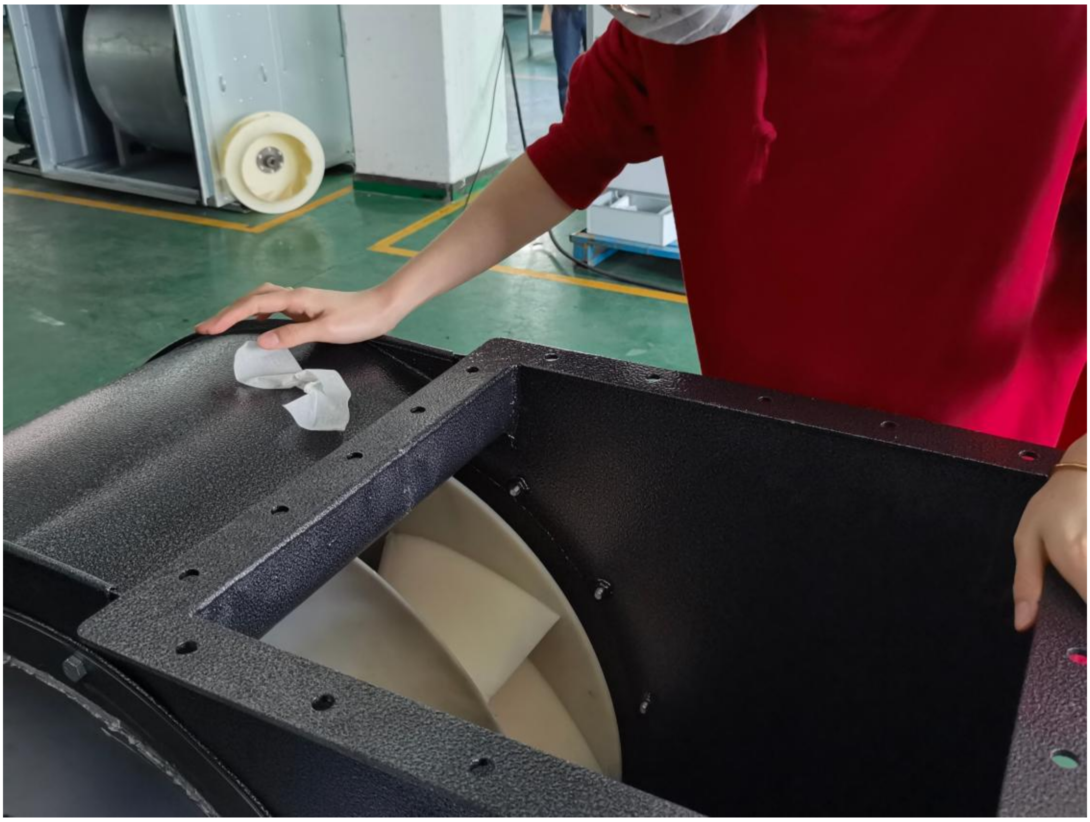

Figure S1 The test fan with a 3D-printed centrifugal impeller.

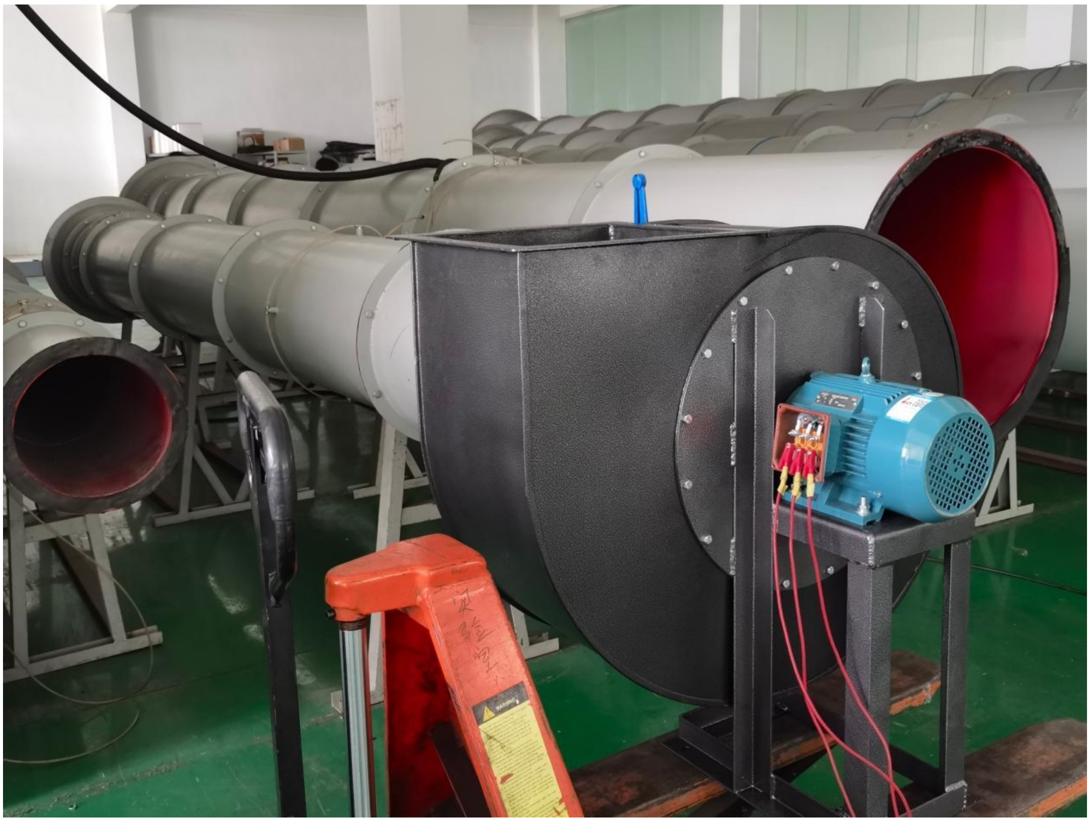

Figure S2. The experimental platform with the test fan installed.
